# Designing and conducting interventional trials with passive-sensing applications on patient-owned smartphones: challenges and recommendations from the BD4QoL study

**DOI:** 10.3389/fdgth.2026.1831734

**Published:** 2026-07-13

**Authors:** Francesco Giuliani, Laura Lopez-Perez, Franco Mercalli, Tessa Fulton-Lieuw, Claudia Vener, Francesco Ricciardi, Michela Falcone, Antonio Mangiacotti, Stefano Cavalieri, Antonello Manocchio, Elena Martinelli, Luca Maria Lacerenza, Chiara Copelli, Despina Elizabeth Filippidou, Aritz Bilbao, Aitor Almeida, Hisham Mehanna, Giuseppe Fico, Lisa Licitra

**Affiliations:** 1 Innovation and Research Unit, Fondazione IRCCS Casa Sollievo Della Sofferenza, San Giovanni Rotondo, Italy; 2 Universidad Politécnica de Madrid-Life Supporting Technologies Research Group, ETSIT, Madrid, Spain; 3 MultiMed Engineers srl, Parma, Italy; 4 Institute for Head and Neck Studies and Education (InHANSE), University of Birmingham, Birmingham, United Kingdom; 5 Epidemiology and Prevention Unit, Department of Epidemiology and Data Science, Fondazione IRCCS Istituto Nazionale dei Tumori, Milan, Italy; 6 Internal Medicine Unit, Fondazione IRCCS Casa Sollievo Della Sofferenza, San Giovanni Rotondo, Italy; 7 Department of Oncology and Hemato-oncology, University of Milan, Milan, Italy; 8 Head and Neck Medical Oncology Department, Fondazione IRCCS Istituto Nazionale dei Tumori, Milano, Italy; 9 Maxillo-Facial Surgery, Fondazione IRCCS Casa Sollievo Della Sofferenza, San Giovanni Rotondo, Italy; 10 Maxillo-Facial Surgery, Interdisciplinary Department of Medicine, University of Bari “Aldo Moro”, Bari, Italy; 11 Information Technology Programme Management Office, DOTSOFT, Thessaloniki, Greece; 12 DeustoTech Institute of Technology, University of Deusto, Bilbao, Spain

**Keywords:** digital health interventions, head and neck cancer, mobile health, quality of life, randomized clinical trials, real world data

## Abstract

Digital health technologies offer novel avenues to support cancer survivorship, but their evaluation in clinical trials poses specific methodological and technological challenges. This policy brief paper reports key lessons stemming from the experience of the BD4QoL multicenter randomized clinical trial (RCT), in which an apps' ecosystem for monitoring the quality of life of a multinational cohort of head and neck cancer survivors was implemented. The project deployed a digital platform on patients' own smartphones to gather behavioral data and patient-reported outcomes during post-treatment follow-up. Drawing from this experience, we describe challenges encountered during trial execution including device heterogeneity, reliance on third party apps, regulatory concerns and methodological barriers, and propose actionable advice to support the rigorous and sustainable implementation of digital health intervention for the next generation of RWD-guided RCTs. In particular, this paper examines the challenges of conducting a multicenter randomized trial based on passive sensing and a Bring Your Own Device (BYOD) deployment approach.

## 1 Introduction

Rapidly evolving digital health technologies are changing in many respects how healthcare is delivered to patients worldwide. Mobile applications and wearable connected devices now enable continuous, real-time collection of patient outcomes gathering data ranging from vital parameters to behavioral patterns (1). Digital innovations emerged initially in medicine as tools for easing communication and monitoring and, nowadays, enabled by artificial intelligence (AI) algorithms (2), are evolving as means to deliver sophisticated interventions (as in the case of digital therapeutics), which are valuable in terms of decision support for clinicians and to promote behavior change in patients. This is true for all medical fields and especially for oncology, where digital technologies enable the extension of care services beyond traditional clinical settings allowing more continuous, personalized, and patient-centered approaches. Head and neck cancer (HNC) survivors represent a group of patients that can greatly benefit from the advances of digital technologies due to disease severity and oncological treatments leading to significant consequences in daily life. HNC survivors often experience long-lasting functional, psychological, and social impairments, due to their compromised ability to speak, swallow, or breathe, or their modified appearance after surgery, which can lead to detrimental socioeconomical effects and patients' self-esteem decline.

In this context, we report the experience of the Randomized Clinical Trial (RCT) stemmed from the BD4QoL project (3, 4), financed by the European Union in the Horizon 2020 research and innovation framework, which developed digital tools for measuring and monitoring Quality of Life (QoL) in HNC survivors.

Digital applications in this field are increasingly claiming clinical use and, for this reason, rigorous investigation and validation (5) in well-regulated ethical and legal frameworks are required. Demonstrating efficacy, safety, and usability requires well-designed interventional studies, meeting standards comparable to those applied to pharmacological or medical device-based interventions. However, digital health interventions introduce methodological and operational complexities that challenge conventional trial designs, including issues related to application deployment, technical maintenance, data collection continuity, user engagement, and integration into clinical workflows (6, 7), as we will discuss in the next sections.

Importantly, interventional trials that use digital applications are not only a way to assess safety and efficacy of the developed solutions, but also an opportunity to generate critical insights on possible implementation pitfalls and to design appropriate corrective actions (8). Issues related to patient recruitment and adherence, technical reliability, ethical concerns, and data integration into clinical workflows frequently emerge during actual trial execution. Capturing these insights is important to orient policy makers, for their possible impact in regulatory pathways, and to promote best practices in digital health research.

While existing literature has already described operational challenges in oncology mHealth trials, less attention has been devoted to the specific intersection of passive sensing and BYOD (Bring Your Own Device) strategies. We address this gap by leveraging the field experience gained during the RCT conducted within the BD4QoL project. This analysis constitutes the distinct contribution of our work to the field.

## 2 Related work and contribution of the present study

A growing body of literature has explored the use of patient-owned mobile technologies and passive sensing strategies to support remote monitoring and longitudinal data collection in patient cohorts. For example, studies adopting this approach in various medical specialties such as oncology (9), Parkinson's disease (10) and multiple sclerosis (11) report that using patients' own smartphones in real-world settings is feasible for delivering services like remote symptom tracking, smartphone-based passive sensing of QoL and clinical outcomes, and disease self-management.

These studies support the idea that consumer mobile devices can enhance clinical observation with further data, added to the ones recorded in traditional healthcare settings, while reducing the burden associated with dedicated monitoring equipment.

At the same time, this literature concordantly reports relevant methodological and operational constraints. Common described barriers include variability in patients' ability to engage with digital technologies (12), possible exclusion of patients using specific smartphone devices or operating systems together with possible limitations in continuity of data collection and standardization (13).

The experience of the i-Prognosis project (14, 15), although focused on Parkinson's disease rather than oncology, provides relevant insights on these points. The objective of this project was to leverage behavioral markers derived from mobile devices to detect the onset of the symptoms of Parkinson Disease. In this case, the researchers reported regulatory, ethical and technical hurdles related, for example, to possible data leakage from the other apps installed on patients' smartphones as relevant challenges they faced (16) together with the need to clinically validate the alarms generated by the algorithms (17). Similar challenges were reported in research initiatives like FAITH (18), ASCAPE (19), REBECCA (20), LifeChamps (21) and ONCORELIEF (22), which faced the challenges of adopting a technology based approach to monitor QoL of cancer survivors.

A particularly relevant contribution for better positioning this work is the recent paper by Stuijt et al. (23), which synthesized lessons learned in three mHealth trials in the field of oncology that leverage on the Trial@home platform as a tool to collect digital endpoints. Beyond a focus on demonstrating technical feasibility, the authors analyze the recurrent challenges occurring in these studies. They show how participant adherence, operational burden, data completeness, and execution-related factors substantially influence the successful evaluation of mobile health technologies across trial planning, design and study execution.

The present work is complementary rather than overlapping with this study and the others cited above. While Stuijt et al. (23) adopt a cross-study perspective to derive general lessons across heterogeneous mHealth experiences leveraging on the use of wearable or nonwearable connected devices, our contribution is rooted in the execution of a single multicenter RCT, therefore providing a more operational perspective on the specific aspects pertaining to a BYOD deployment approach, in which passive sensing technologies leverage on patients' personal smartphones to continuously collect behavioral and QoL related information over extended follow-up periods.

The distinction is relevant because maintaining continuous passive sensing in patient-owned devices introduces methodological and infrastructural challenges that are less represented in the existing literature. Furthermore, the BD4QoL experience provides insight into the interaction between conventional RCT assumptions and the dynamic evolution of consumer digital applications and ecosystems. Our findings highlight practical challenges associated with maintaining longitudinal data continuity despite evolving mobile operating systems, third-party app dependencies, heterogeneous smartphone configurations, and sustained participant engagement during long-term follow-up. By documenting these operational aspects and translating them into implementation-oriented recommendations, the present work extends previous literature toward a more detailed understanding of how such digital interventions can be sustained in real-world conditions.

## 3 Background: the BD4QoL study

BD4QoL (Big Data for Quality of Life) (3, 4) was a European large-scale, multidisciplinary initiative aimed at improving QoL of HNC survivors using AI and unobtrusive digital technologies. Adult patients treated with curative intent for non-metastatic Head and Neck Squamous Cell Carcinoma (HNSCC) across different subsites, as well as selected non-metastatic Salivary Gland Cancers (SGC), disease-free at enrollment, were recruited into the prospective RCT generated by the project. The patients were followed up in the post-treatment phase, a period commonly characterized by diminishing QoL due to the persistency of therapies-related toxicities, functional impairments, and psychosocial sequelae.

This RCT (4) enrolled 410 HNC survivors across multiple centres in Italy (Fondazione IRCCS Istituto Nazionale Tumori, Milan—Fondazione IRCSS Casa Sollievo della Sofferenza, San Giovanni Rotondo—Azienda Ospedaliero Universitaria Consorziale Policlinico di Bari) and the United Kingdom (University Hospitals Birmingham NHS Foundation Trust). Participants were randomized in a 1:2 ratio to either standard follow-up (control arm) or to a digital intervention based on the use of the BD4QoL platform (intervention arm). The intervention consisted of the deployment of a smartphone-based monitoring ecosystem directly on participants' personal Android devices with a BYOD approach.

Both the intervention and control group completed longitudinal assessments using validated patient-reported outcome measures (PROMs), including EORTC QLQ-C30, EORTC QLQ-HN43, EQ-5D-5L, and CBI-B questionnaires at baseline (enrollment date) and every 6 months till the end of the established 24-month follow-up period.

According to the study protocol, the primary objective was to demonstrate a significant reduction in the proportion of patients in the intervention group who experienced a clinically meaningful deterioration in quality of life (QoL) when compared with the patients in the control arm. Clinically meaningful deterioration was defined as a decrease of at least 10 points in the EORTC QLQ-C30 global health status score. A side objective of the study was to leverage digital technologies to identify and capture traits of potentially actionable QoL items, which are often insufficiently captured by standard of care clinical follow-up.

The core components of the BD4QoL digital ecosystem delivered to the patients of the intervention arm are: (1) a foreground app (the BD4QoL F-App) operating the collection of continuous behavioral proxies of health status, read from the smartphone sensors (physical behavior, social behavior, sleep); (2) a web tool for the periodical collection of validated QoL questionnaires; (3) a patient-facing dashboard app (the BD4QoL M-App) hosted in the smartphone that allowed patients to visualize their data trends over time, including a chatbot interacting with patients through a text interface to offer symptoms management support and to motivate towards physical activity. Instead, the application of the BD4QoL ecosystem developed for the physicians are: (4) Point of Care (PoC) tool that allowed to monitor patient collected data, receive smart alerts, and detect relevant deviations from baseline behaviors or significant QoL variations; and (5) an electronic Case Report Form (eCRF) which collected demographic and clinical data [using REDCap (24)].

The collected data were integrated within a secure architecture designed to support the monitoring of longitudinal QoL trajectories. Advanced machine learning and time-series modelling techniques were used to identify early signals of clinically meaningful QoL deterioration.

The BD4QoL consortium adopted an Android-only strategy for smartphone-based passive sensing as Android provided access to a wider range of passive sensing data streams required by the study protocol, while Apple-based smartphones posed substantial restrictions on background sensing due for example to the impossibility for third-party apps to collect phone and SMS (Short Message Service) logs. Moreover, location-related measurements on iOS were available at lower temporal granularity (approximately one sample every 15 min) compared with the 1-minute sampling interval achievable on Android devices (25). This difference was expected to impact the performance of the semantic location recognition algorithms used to infer visited places and mobility patterns, potentially reducing quality of behavioral characterization. Therefore, lack of availability of an Android OS in patient's smartphone was an exclusion criterion for participation in the study. This design choice may have introduced selection bias toward Android users, as it potentially led to the exclusion of patients with different socioeconomic profiles.

Beyond its clinical objectives, the BD4QoL RCT addressed policy-relevant issues linked to cancer survivorship demonstrating how digital platforms can complement traditional survivors' clinical management by generating high-quality clinical evidence to be integrated into routine oncology care.

The technology deployment workflow was implemented through a standardized researcher-assisted onboarding process, conducted at the enrollment visit on the participant's own Android smartphone [required Operating System (OS) version ≥7.0]. At first, the research staff registered the participant into the study eCRF to obtain the unequivocal study identifier number (ID), and then confirmed the participant's digital identity through the Questionnaire Tool (web-app). This initial step allowed the researchers to facilitate the first access and trigger a secure password setup link, ensuring that the participant's credentials were correctly synchronized with the study database from the outset. Following this, a three-fold app installation process was performed for the patients randomized in the interventional arm: first, Google Fit was configured as the primary middleware for physical activity aggregation; the BD4QoL F-App was then deployed to maintain persistent sensor-to-server communication; and finally, the BD4QoL M-App was installed to provide the user interface for daily monitoring and chatbot interaction. An impactful action during this phase was modifying smartphone settings that, in order to preserve battery life, automatically restrict the activity of background apps. In this case, researchers configured smartphone settings to ensure that the study applications could operate continuously and access the location and movement sensors needed throughout the monitoring period. This was a key measure to prevent interruptions in data collection caused by automatic power-saving functions.

The technical setup was completed with face-to-face training sessions in which researchers provided a general overview of the BD4QoL apps functionalities to the patients. Detailed information on the deployment of the BD4QoL mobile infrastructure including installation, account setup, permission configuration, cross-app integration, post-installation validation, quality assurance procedures, and technical support actions to ensure continuous data collection, is reported in the Supplementary Material. Research staff utilized the Point of Care (PoC) tool dashboard to monitor data synchronization status and in case a lack of data transmission was detected lasting for more than five consecutive days, a standardized troubleshooting protocol was initiated. This involved direct outreach to the participant to provide remote assistance for common issues, such as accidental sensor deactivation or OS-initiated application lock. Moreover, during each on-site patients' follow up visit, the study staff directly checked the BD4QoL apps settings to see if everything was in place and well set up.

## 4 Challenges and recommendations for BYOD passive-sensing trials

Assessing digital health interventions in the framework of a clinical trial setting raises a set of issues that extend beyond those typically faced in clinical research that investigates the effect of a drug or a medical device (26).

Despite the literature in the field reports that digital health interventions are generally feasible and in many cases effective (9, 27, 28), running a RCT fueled with data collected through the last up-to-date digital solutions may be challenging, considering that the technical feasibility does not eliminate the frictions that could arise during patient use of the technologies, specifically under prolonged real-world deployment conditions. These concerns become more relevant when interventions rely on patient-owned smartphones and continuous passive sensing, as participants maintain control over device configuration while the underlying software ecosystems continue to evolve during study execution.

The challenges we faced in the BD4QoL study align with the broader observations reported in the previous studies, particularly regarding the complexities linked to user acceptance of technology and the predictable variability in user behavior, both of which can influence patient adherence.

In addition to these aspects, in the following we highlight how the methodological assumptions rooted in medical research relate to real-world studies, especially in light of the fast-paced evolution of digital technologies. We describe in greater detail these challenges and how we faced them in BD4QoL in the next subsections.

### 4.1 Methodological and technological constraints

The design and implementation of trials in clinical sciences heavily relies on sound methodological conduct, established in foundational literature articles (29, 30) and widely adopted as a guiding value in clinical care. One of the core tenets is that, when designing a prospective interventional trial, the single agent being tested (usually a drug or a device) must be immutable in principle across the trial duration. In the BD4QoL RCT, we faced the difficulties of a clinical trial intervention in which the active principle (the BD4QoL app ecosystem) was subject to various modifications and updates and lived in a fast-evolving OS/app/cloud ecosystem. As examples, we faced the challenge of dealing with: (1) the discontinuation of the Google health-data integration service, which the BD4QoL apps originally relied on to collect physical activity data such as step counts, while the clinical trial was underway, obliging us to develop custom software components to gather steps data directly from the smartphone sensors (a complete account of the actions and remediation workflow we performed to face this issue is detailed in the Supplementary Material S6); (2) unanticipated restrictions on permission management introduced in newer Android OS versions; and (3) programming bugs reported by the users after the apps had been delivered to them, together with app upgrades for patients enrolled before the release of the newest versions. These issues illustrate an often-necessary dependency on third-party platforms, in which researchers, study coordinators, study nurses, technologists and dedicated personnel often have zero control, with unexpected impacts in terms of homogeneity and quality of the study.

The impact of these technological challenges became evident when evaluating the continuity of passive sensing during trial execution (Table 1). Among 248 participants randomized to the intervention arm, passive sensing data availability was classified and analyzed using two proxy indicators representative of the overall functioning of the mobile infrastructure: daily step counts (collected via the F-App) and GPS data (collected through the background sensing component). Based on predefined thresholds, 106 participants (42.7%) achieved a high level of data availability (≥50% completeness for both indicators), 44 (17.7%) medium-quality availability (30%–50%), and 98 (39.5%) low-quality availability (<30%). These findings indicate that maintaining reliable passive sensing over prolonged periods in patient-owned smartphones remains challenging despite accurate onboarding, monitoring procedures, and technical support. We also observed a clear mismatch between the rapid pace at which digital ecosystems evolve and the timescales required to conduct RCTs (31). Despite the relatively short duration of the BD4QoL RCT, the study was still exposed to changes in operating systems, application behavior, and software dependencies that required continuous adaptations. These needs were reflected by the software maintenance activity performed during the study period when multiple releases of both the BD4QoL F-App and M-App were required to maintain operational continuity, introducing fixes for background process persistence, permission management for unused applications, chatbot session handling, notification workflows, and compatibility with evolving external services. These changes highlight how digital interventions may require continuous maintenance even after formal deployment, challenging the methodological assumption that interventions should remain technically unchanged throughout trial execution. Examples of troubleshooting procedures and reconfiguration workflows that we performed are reported in Supplementary Material.

**Table 1:** Selected indicators concerning the BD4QoL RCT that illustrate challenges associated with maintaining continuous passive sensing data on patient-owned smartphones. Passive sensing availability was based on completeness of GPS and daily step data streams, respectively selected as proxy indicators for the operational status of the background and foreground components.

Indicator	Value
Participants in intervention arm	248
Good passive sensing data availability (Stable acquisition of daily steps and GPS data)	106 (42.7%)
Medium passive sensing data availability (Partial continuity of data collection)	44 (17.7%)
Low passive sensing data availability (Significant limitations in continuity of data gathering)	98 (39.5%)
Main app releases during study	8
Foreground app releases during study	8

Furthermore, one of the initial study design challenges was whether or not a dedicated smart device should be used to track daily activities and the digital biomarkers of interest (32, 33). We opted to develop an information ecosystem that would leverage apps residing in the patients' own smartphones instead of a dedicated device. This approach was preferred to underscore the importance of usability, unobtrusiveness, sustainability, and to relieve a possible stigma for the patient wearing a “cancer-related device”. Other studies adopted the same approach (9). However, this design choice led to some pitfalls in terms of standardization of the measurements and of ease in applying technical fixes to the apps, given that we did not pass through official app stores for app updates. Another relevant aspect was the possible uninstallation of the BD4QoL apps, or the modification of the BD4QoL apps permission/background settings by the users that considered them battery draining. This behavior occurred in many cases after several weeks of routine smartphone use after enrollment. In the BD4QoL case, the major source of battery consumption dealt with the background app, which was responsible of collecting all the data read from the smartphone sensors (e.g., accelerometer, GPS, call logs and app usage logs, etc.). Additional challenges concerned participants' compliance with the technical usage recommendations, like not switching off the smartphone, not removing or modifying background permissions, and maintaining the battery level above 20%. Finally, we can also report that few users changed their smartphone during the 24-month study period, which required reinstalling the applications on newer devices.

#### 4.1.1 Implications for researchers and funders

In light of the aforementioned challenges, we suggest that system architecture and deployment strategies should be planned with practical real-world implementation at the forefront, while being optimized for a clinical trial. This includes anticipating impactful smartphone OS updates or possible modifications of third-party services, which may be critical for system operations, or to ensure reproducibility of the developed innovation beyond the experimental setting. In addition, remote configuration of the systems should be preferred. When remote modifications cannot be implemented, notification or alert systems may not be sufficient to ensure effective changes in real-world conditions, since patients retain control of their devices. This can lead to increased patient contacts or additional time during in-person visits to configure the app. Therefore, we suggest a flexible planning of the study, in a way that it can accommodate for tool changes and possibly to host the developed applications in verified mobile app stores. Beyond the aspects specifically pertaining to smartphone-based interventions, more in general, because experimental conditions in digital interventions are partially controllable, researchers should consider adaptive methodologies for the implementation of clinical studies. Approaches like including extensive beta testing before participant involvement, internal testing workshops to deal with different developer components and third-party services, more flexible constraints on trial execution, or a hybrid design trial (34–38) may help address these challenges. In particular, hybrid trials, which integrate elements of clinical effectiveness research with implementation research, can better capture the effectiveness of the intervention in real-world settings. These approaches can also align the generation of evidence with the dynamic nature of digital health technologies. For funders this can translate into granting a budget for continuous software maintenance during trial execution. Furthermore, to successfully face the challenges of adopting digital health technologies in clinical trials, researchers must look toward the principles of Implementation Science (39). By mapping barriers through the use of determinant frameworks like the Consolidated Framework for Implementation Research (CFIR) (40), deploying targeted implementation strategies such as the ERIC (Expert Recommendations for Implementing Change) taxonomy (41), and measuring specific implementation outcomes, like feasibility and acceptability, for instance using Proctor's Taxonomy of Implementation Outcomes (42), researchers can ensure that digital innovations are not only clinically effective but also practically adoptable and equitable for diverse patient populations.

Furthermore, digital health solutions should be developed with the final users being fully involved in the development cycle. Methodologies like Agile development (43), that actively involve stakeholders (patients, informal and formal caregivers), can help ensure usability, raise user experience, while allowing a continuous refinement of the solution which can lead to true user defined technologies.

In addition, to grant a real impact to digital solutions the interventions and technical activities should be designed with interoperability in mind (44). Integration with local Health Information Systems and, where feasible, with national Electronic Health Records on a larger scale is essential to support future translation of these innovations into routine care. We recommend including these aspects in the design phase and soliciting the engagement of institutional Information Technology (IT) infrastructure to ensure full compliance with established interoperability standards.

Finally, it is important to evaluate during the initial stages whether the developed systems qualify as medical devices under the EU Medical Device Regulation or US Food and Drug Administration framework or are high-risk systems according to regulations concerning AI-based applications (e.g., EU AI Act). Clarifying the regulatory status before the development of the applications allows appropriate planning for the needed certification or assessment steps, especially when evaluating post-project impact. This is mandatory if the developed innovations go beyond the prototyping phase to reach the market. Such aspects are gaining relevance in light of the rapidly expanding market of digital therapeutics across multiple medical specialties, where regulatory compliance represents a key prerequisite for commercialization and clinical adoption.

### 4.2 User-Centered trial design and engagement barriers

Beyond methodological and technological challenges, a digital intervention trial can generate significant issues related to user-centered design, patient engagement, and trust. We also faced such issues in the BD4QoL project and they are common in digital interventions deployed with a BYOD approach (see Table 2). A first critical problem concerned the development of user interfaces and interaction flows to ensure unobtrusiveness. As reported in the literature (45, 46) differences in age, digital literacy, cultural background, and prior exposure to technology may influence how patients perceive and use digital tools, often resulting in variable engagement with the intervention. We observed that, compared to younger survivors, older participants in the HNC cohort often required more intensive support to manage background permissions and app settings (for example, when the smartphone OS needs to be updated, or when the BD4QoL apps' settings are reset by “external factors” like prolonged smartphone switching off, prolonged battery low level and activation of energy saving mode). Closely related to this is the challenge of maintaining usability in a real-world clinical setting. We opted for simple interfaces avoiding excessive notifications, which could have increased the cognitive burden, potentially reducing adherence. We also experienced in few patients a sense of distrust stemming from limited familiarity with the digital world, along with a fear of possibly being monitored or concerns that automated systems might eventually reduce appropriate human care. Furthermore, sustained engagement over long trial durations is challenging when the perceived burden of using the technology outweighs the perceived benefits. Mistrust regarding data privacy was a concrete obstacle during the recruitment process. Some patients were reluctant to share sensitive health or behavioral data when they perceived uncertainty about how their data would be reused. These factors directly contributed to attrition during the trial, as also reported in another study (47). Despite these concerns, the BD4QoL RCT experienced a lower than expected dropout rate. While we estimated, as reported in the clinical protocol, a 20% dropout rate (which was assumed as one of the inputs to compute the study statistical power), the observed rate was only 6.34%. Although causal inference cannot be established, the observed lower than expected dropout rate suggests that structured communication strategies on data confidentiality may have contributed to participant retention in the study.

**Table 2:** Main challenges identified during the design and conduct of the BD4QoL project, together with corresponding mitigation strategies derived from the literature and from practical experience. The table highlights challenges specifically associated with patient-owned smartphone deployment (BYOD) in digital health trials, highlighting the trial phase in which each identified challenge manifests.

Challenge	Potential impact	Mitigation strategy	BYOD specific	Trial phase
Methodological & technical challenges				
Dependence on third-party APIs and OS updates	May compromise longitudinal data consistency	Use modular architectures and contingency plans for API/OS changes		Design, Intervention
Device heterogeneity across patients' smartphones	Reduced data standardization and sensor consistency (Variability in device hardware, permission management, and sensors)	Define minimum technical requirements and test across multiple devices monitoring compatibility during trial execution	✓	Design, Recruitment, Intervention
Distribution of updates outside official app stores	Delayed bug resolution and increased support burden	Prefer verified app distribution channels and implement remote update mechanisms	✓	Intervention
Battery consumption from continuous sensing (background processes)	Missing data due to app uninstallations or permission changes	Optimize sensing frequency and monitor energy consumption during pilot testing or in a pre-trial sandbox environment	✓	Design, Intervention
Study conduct				
Patients changing smartphone models during the study	Interruptions in longitudinal monitoring and need to reinstall/reconfigure	Define replacement and reconfiguration procedures to minimize data losses	✓	Intervention
High workload for research staff managing technical issues	Increased burden on clinical sites affecting trial efficiency	Allocate dedicated technical support and staff training for troubleshooting		Recruitment, Intervention
Methodological assumptions and real-world conditions				
Tension between rigorous trial conduct and evolving digital app ecosystems	Software updates or platform changes may conflict with intervention stability	Differentiate major clinical updates from minor technical updates, less impactful on the clinical study		Design, Intervention
Limited control over real-world experimental conditions	Reduced reproducibility and standardization	Adopt pragmatic or hybrid trial designs incorporating implementation science principles		Design
Trade-off between ecological validity and measurement standardization	BYOD approach improves realism but lowers measurement consistency	Align endpoints with expected data quality and device strategy weighing BYOD against a provisioned-device strategy	✓	Design
User-centered design & engagement				
Variable digital literacy among participants	Reduced onboarding and adherence/retention	Provide training materials and involve patients in co-design activities		Recruitment, Intervention
Cognitive burden from multiple apps or notifications or complex interfaces	Reduced sustained engagement	Simplify interfaces and prioritize unobtrusive design		Design, Intervention
Privacy & regulatory				
Data privacy risks on personal smartphones hosting multiple apps	Potential unintended data exposure or misuse	Conduct DPIAs and communicate safeguards transparently to participants	✓	Design, Recruitment, Intervention
Surveillance concerns	Reduced trust and willingness to participate	Provide active and simplified communication about safeguards		Recruitment, Intervention
Uncertainty regarding appropriate regulatory classification of the apps	Potential delays in approval and adoption	Align development with MDR, FDA, and/or AI Act requirements		Design, Post-trial
Post-project sustainability				
Limited interoperability with Healthcare Information Systems	Reduced post-trial integration into routine clinical care	Adopt interoperability standards and involve institutional IT staff in system design		Design, Post-trial

#### 4.2.1 Implications for researchers and funders

To address these difficulties, we propose a set of recommendations that emphasize the importance of user-centered trial design and conduct.

First, researchers should consider socioeconomic disparities among study participants, which are reflected in different levels of technology adoption and smartphones with different price ranges and performance. One consideration may be to set up a recruitment strategy that randomizes by these factors to ensure representativeness of different digital skill profiles. Another factor is understanding digital literacy as an important driver of adoption, and how it may influence adherence. In addition, one way to enable the participation of patients with incompatible smartphones would be to provide them with newer devices that, with their consent, could serve as their primary personal smartphones during the trial. This option must be considered only as a last-resort measure, since the goal of ensuring the inclusion of participants with diverse digital profiles must be balanced against the potential impact that using an unfamiliar device may have on participant engagement and adherence (see the fourth point below), which is the reason why we adopted a BYOD approach in BD4QoL.

Second, researchers should adopt clear and simplified communication with study participants to inform them about all the measures in place to ensure a cybersecure approach to data privacy and management, considering that every clinical study must possess a data management plan, a formal DPIA (Data Privacy Impact Assessment) evaluation, informed consents and privacy information sheets. A well-structured communication plan that incorporates a patient-centered approach, which can be easily delivered by participating centers, may help support recruitment into trials with digital technologies. According to the approach recommended by privacy regulations, careful consideration should be given to the design of a robust data management plan that can guide the development of data related activities including, after the end of the project, the reuse of the generated datasets for the benefit of scientific progress.

Third, it is crucial to clearly present to study participants the possible tangible benefits of their participation in the trial (e.g., better health status and lifestyle monitoring) and the broader value of the study for advancing research in the specific field, leading to improved care for future patients. These actions can mitigate attrition over time.

Fourth, we can testify the feasibility of studies in which digital interventions leverage on the smartphone already owned by patients, avoiding the need to wear, recharge, or manage multiple dedicated devices. This approach lowers participation barriers, reduces burden, and helps integrate the intervention into daily life, as patients are already familiar with their own devices and their interfaces, reducing also the need for additional training on how the devices work. Even if we recommend this approach, adopted by us in BD4QoL, it is straightforward that it will lead to significant technical burden (1) for the team of software developers; (2) for the researchers dealing with continuous checking of apps' settings on patient smartphones; and (3) for the data analysis team, which will possibly have fewer, or only indirect variables, to consider with respect to the ones obtainable when using dedicated devices. This approach can produce a burden for the patients too, as reported in Section 4.1, specifically in terms of battery consumption of the installed apps operating in background and in charge of collecting the data generated by the smartphone sensors.

Furthermore, we suggest to hold training and engagement workshops with a cohort of study subjects pre-enrollment, or even during enrollment visits, with the direct involvement of the team of software developers.

Finally, simplicity should be a guiding principle in technological design as minimizing dependencies on multiple applications, accounts, or services can meaningfully improve reliability and usability.

Table 2 summarizes the main challenges encountered in the design and the conduct of the BD4QoL RCT and maps them to trial phases and actionable recommendations from the literature and from our experience, distinguishing issues that are specific to patient-owned smartphone deployment and those that apply to digital health trials in general.

### 4.3 Limitations of the BD4QoL study

The experience gained in the BD4QoL project provides actionable insights into the implementation of smartphone based digital interventions within a RCT even though a series of limitations should be considered when interpreting the observations and recommendations reported in this paper.

As a first point, the study adopted an Android-only strategy to enable the passive sensing functionalities required by the intervention. As previously discussed, this choice was due to technical constraints related to access to smartphone generated data and background sensing capabilities. However, excluding users of non-Android operating systems may have reduced the representativeness of the HNC survivors' population, limiting the generalizability of the findings to broader populations with different device preferences or socioeconomic characteristics.

Secondly, a limitation concerns the technical architecture of the BD4QoL ecosystem. The intervention relied on multiple interconnected software components rather than a single application environment. This modular approach facilitated the separation of functions and independent component evolution but increased deployment complexity, configuration burden, and the number of potential failure points.

Furthermore, the BD4QoL digital ecosystem relied by design on third-party applications and services for some key functionalities, thus introducing an external source of variability that remained outside direct control of the consortium. Changes in third-party policies, software upgrades, or discontinuation of services required technical adaptations during trial execution and represented a challenge for reproducibility and long-term sustainability.

From a more methodological perspective, although the availability of GPS and step data was used as a proxy to estimate operational continuity of the infrastructure, missing observations could not always be unequivocally attributed to technical causes (e.g., permission set up, software malfunctioning, operating system restrictions, connectivity issues, or device replacement) vs. participant-related behaviors such as reduced engagement or modifications in smartphone use habits. Consequently, data completeness should not be interpreted exclusively as a measure of patient adherence.

Finally, device changes by the patients introduced additional variability related to hardware characteristics, operating system configurations, and application reinstallation procedures, thus affecting continuity of data collection.

These limitations notwithstanding, we believe that documenting such constraints represents an important contribution of the present work, as they reflect practical issues likely to emerge in future clinical studies adopting strategies based on patient-owned smartphones and passive sensing of behavioural traits.

## 5 Conclusions

Despite the challenges, the experience of the BD4QoL RCT demonstrates that digital technology interventions for vulnerable oncology patients are feasible, even when relying on apps deployed on patients' smartphones. However, to achieve better results researchers must recognize that traditional approaches to evidence generation should be adapted to comply with the very dynamic nature of digital technologies and to the human factors influencing adoption and adherence. Digital health interventions should be considered as highly dynamic across the entire duration of the trial, and funding and regulatory approval bodies must accept and support the process of iterative updates through the trial lifecycle, distinguishing core clinical changes and minor functional updates that typically arise from the digital ecosystem in which the applications operate. The proposed recommendations can contribute to the successful design and evaluation of digital solutions in healthcare, ensuring that these solutions can extend beyond the prototyping phase toward sustainable integration in real-world clinical care pathways.
