# Supplementary material for: Designing and conducting interventional trials with passive-sensing applications on patient-owned smartphones: challenges and recommendations from the BD4QoL study
Source: Front Digit Health. 2026 Jul 13;8:1831734. doi: 10.3389/fdgth.2026.1831734 (PMC13402479; doi:10.3389/fdgth.2026.1831734)
Supplement: Supplementary file 1 [file Datasheet1.pdf]

Supplementary Material

1 Supplementary Data - Installation and Configuration of the BD4QoL Mobile Tools

The installation of the BD4QoL ecosystem on participants' devices followed a standardized multi-step protocol to ensure consistent data collection and app stability across different Android versions and manufacturers.

– *Google Fit Integration*

Initially, participants installed the Google Fit application from the Play Store on their personal mobile devices. The setup required logging in with a preferred Google account and completing the "About you" profile section. Crucially, the "Automatic track of activities" feature was enabled to allow physical activity monitoring. For devices running Android 11 or higher, the "Remove permissions if the app is unused" toggle was disabled. Furthermore, for users with OS ≥ 10 on specific hardware (Xiaomi, Oppo, Vivo, Huawei, or TCL), the "Autostart" permission was activated within the application settings to prevent background service termination.

– *Portal Access and Credential Management*

Participants accessed the study's dedicated Participant Portal via a web browser to navigate the "Patient Information" and "App Installation" sections. Before app deployment, a mandatory security reset was performed for the Questionnaire web app. Using their default ID, participants were required to link a personal email account, confirm it via their inbox, and create a new personalized password through the reset link provided.

– *Foreground App (F-App) Deployment*

The Foreground App was downloaded as an APK directly from the Participant Portal. After selecting the language and logging in with the newly updated credentials, participants initiated the "Start Data Collection" phase. This required granting several critical system permissions:

- General permissions for data access.
- Disabling battery usage optimization to allow persistent background activity.

- For OS ≥ 11, ensuring "Remove permissions if app is unused" was set to OFF.
- For OS ≥ 10 (Xiaomi, Oppo, Vivo, Huawei), enabling the "Autostart" option.
- For TCL devices (OS ≥ 10), a specific procedure was followed using the "Smart Manager" app: disabling the "Automatically manage" toggle and manually enabling "Autostart" for the Foreground App.

– *Main App (M-App) Installation and Inter-App Linkage*

Finally, the BD4QoL Main App was installed via APK. After logging in with the reset credentials, participants finalized the setup by authorizing:

- Battery optimization bypass.
- Full permissions for calls and SMS monitoring.
- API Linkage: users clicked the Google Fit logo within the Main App to authorize the digital handshake between the two applications.
- Manufacturer-Specific Settings: the same Autostart and permission retention protocols (OS ≥ 11 and OS ≥ 10 for Xiaomi, Oppo, Vivo, Huawei, and TCL) applied during the Foreground App setup were strictly replicated to ensure the Main App's operational continuity.

2 Implementation and Validation Protocol

To ensure the technical integrity of the data collection infrastructure and the reliability of the monitoring system, a standardized "Same Day Installation Checklist" was developed and applied immediately following the deployment of the software on each participant's device. This protocol is structured into three primary domains: visual verification, functional data validation, and system permission optimization.

– *Initial Setup and Visibility*

The first stage of the protocol confirms the correct installation of the dual-app architecture. Researchers verify the presence and visibility of both the Main application and the Foreground application on the mobile device's interface. A critical checkpoint includes the confirmation of the Foreground app's persistent notification icon, which serves as a primary indicator of the background process's active status.

– *Functional Data Validation and Sensor Integrity*

A rigorous diagnostic procedure was performed through the Foreground application's "Preview Data" feature to ensure high-frequency data acquisition (sampling every 60 seconds). This phase involved:

- Temporal Synchronization: Verifying the accuracy of the offline data timestamp and the correct UTC offset (e.g., +2 hours and 7,200 seconds for the Italian cohort).
- Sensor Calibration: Confirming non-zero, real-time values for a comprehensive suite of sensors, including ambient light (LUX), tri-axial accelerometer (x, y, z coordinates), GPS coordinates, and Wi-Fi connectivity status.
- State Detection: Validating the binary status of the screen sensor (ON/OFF) and the initial calibration of physical activity recognition (e.g., transitioning from an “Unknown” state to “Still”).
- Data Transmission: Executing a “Force Upload” command to verify the end-to-end communication between the mobile device and the central server, confirmed by a “Data Sending Completed” notification.

– *System Optimization and Permission Management*

The final stage of the checklist focuses on preventing OS-level interference with long-term data collection. A centralized “Permission Check” and a “Check System Settings” diagnostic (within the “ME” section of the Main app) were performed to ensure a successful system-wide configuration. To guarantee continuous operation, the following manual adjustments were mandatory:

- Energy Management: Disabling battery optimization for both applications to prevent background process termination.
- Persistence Settings: Deactivating the “Remove permissions and free up space” toggle and enabling the “Autostart” option within the operating system’s application settings.

3 Patient-Led Validation: The “Next Day” quality Audit

To consolidate the reliability of the collected digital biomarkers, the protocol included a secondary validation phase performed directly by the patient on the day following the initial installation. This “Next Day Check” was designed to verify the alignment between the data processed by the Main Application and the user’s actual behavior during the previous 24-hour period.

– *Physical Activity and Mobility Synchronization*

The patient performed a retrospective audit of their mobility patterns through the “Steps” and “My Day” modules. This involves:

- Step Count Verification: Confirming that the total daily steps recorded for the previous day represent a plausible estimation of the user’s physical activity.

- Mobility Classification: Ensuring that the application correctly identified specific activities performed by the patient (e.g., Walking, Running, Cycling, or in-Vehicle) within the “My Day” dashboard, validating the system’s ability to distinguish between different motion intensities.

– *Digital Interaction and Communication Metrics*

A significant portion of the checklist was dedicated to the accuracy of the phone-related passive sensing. Participants were instructed to cross-reference the data displayed in the app with their device’s native logs:

- Telephony and Messaging: Comparing the count of incoming and outcoming calls and SMS messages within the app against the device’s communication history.
- Connectivity and Screen Usage: Validating the accuracy of the recorded Wi-Fi Network Name (SSID) and ensuring the “Screen On/Off” time was correctly visualized.
- Social Engagement: Assessing the plausibility of “Social Application” usage time, which serves as a proxy for digital social interaction, provided all necessary background permissions were active.

– *Human-Computer Interaction (HCI) and Chatbot Functionality*

The final validation step focused on the interactive components of the BD4QoL ecosystem. The patient tested the BiDi Chatbot to ensure seamless bidirectional communication. This included verifying the ability to send and receive messages, as well as confirming the correct triggering and accessibility of chatbot notifications. This step was relevant for ensuring that the primary channel for patient-reported outcomes (PROMs) and psychological support remained functional.

4 Adherence and Data Quality Maintenance

To maximize data continuity and minimize the risk of lack of data transmission, a comprehensive set of "Good Practice Guidelines" (DO’s and DON’Ts) was established for all participants. These guidelines categorized behaviors based on their impact on study integrity, ranging from low to high severity.

– *Device Management and Power Persistence*

The technical cornerstone of the protocol was the continuous operation of the mobile device. Participants were instructed to maintain the smartphone on throughout the day (High Severity) and were encouraged to charge the device frequently to keep the battery level above 20% (Medium Severity). Night-time management was equally critical: while devices remained powered and charging, participants had the option to disable internet connectivity during sleep hours to address personal preferences. Significantly, the protocol required that participants bypass OS-level battery optimizations for the BD4QoL applications to ensure continuous background sensing.

– *Network Connectivity and Synchronization*

Reliable data transmission was facilitated by specific connectivity requirements. A high-priority recommendation was an active internet connection from early morning until noon. While mobile data should ideally remain active throughout the full 24-hour cycle, the reactivation of a data plan after any temporary deactivation was considered a high-priority action to prevent prolonged data gaps.

– *Software Integrity and Usage Patterns*

To ensure the validity of the acquired digital biomarkers, the following strict prohibitions were implemented:

- Application Integrity: The deletion of the Main app, Foreground app, or integrated Google Fit app was discouraged (High Severity). Furthermore, the software should not be installed on multiple devices simultaneously to avoid data duplication or conflict.
- Passive Sensing Fidelity: Participants were advised to use the personal smartphone in which the apps resided as their primary device for calls and social media – rather than PCs or tablets – to capture a representative picture of their digital life. To ensure data purity, sharing the device with third parties was discouraged.
- Permission Stability: Any modification to system permissions, particularly Location Services, should be avoided or discussed with the clinical team to prevent the accidental silencing of sensors.

– *Interaction, Privacy, and Incident Reporting*

Engagement with the Bidi Chatbot was managed through a tiered approach: while spontaneous interaction was encouraged, responding to system-generated notifications within the chatbot session was flagged as a medium-priority task for protocol adherence. For privacy reasons, participants were required to use personal email accounts exclusively within the ecosystem. Finally, a robust communication channel was established for "High Severity" events, requiring immediate notification to clinicians in the event of phone theft, accidental data pause, or the acquisition of a new mobile device.

5 Technical Troubleshooting and Data Integrity Maintenance

To address potential technical failures in passive data collection, a structured troubleshooting protocol was implemented. This systematic approach ensured that any disruption in the flow of digital biomarkers was identified and resolved through a step-by-step diagnostic hierarchy involving three main areas: activity tracking, phone-related sensing, and vendor-specific configurations.

– *Resolution of Activity Tracking Discrepancies (Steps)*

The synchronization between the BD4QoL app ecosystem and Google Fit was a critical dependency for physical activity metrics. In cases where step counts were not reported in the Main app, a sequential audit was performed:

- Application Alignment: Verification of the correct installation and setup of Google Fit, ensuring that both the third-party provider and the BD4QoL app utilized the same Google account.
- Sensor Activation: Confirmation that the "Track your activities" toggle was active within the Google Fit profile settings.
- OS-Level Persistence: Adjusting permission settings to disable the "Remove Permissions for unused apps" feature and enabling "Autostart" for Google Fit to prevent background process termination.

– *Diagnostic Protocol for Passive Phone Data*

For missing communication logs (Calls/SMS) or usage metrics (Social Media/Screen Time), the protocol focused on Android-specific "Usage Access" and "App Permissions."

- System Permissions: A manual check was conducted to ensure that "Usage Access" and specific permissions for telephony and messaging were active.
- Data Validity Monitoring: A "No Valid Days" alert triggers an immediate audit of the Foreground app. If the application was missing or outdated, a fresh installation of the latest software build was performed to restore the reporting mechanism.
- Social Metrics: For social media interaction tracking, the protocol specifically verified that the global usage access permission was granted, as this was the primary source for behavioral social indicators.

– *Vendor-Specific Optimizations and Permission Persistence*

Recognizing the fragmentation of the Android ecosystem, specialized diagnostic procedures were tailored for different smartphone manufacturers to address aggressive background task management:

- Xiaomi and TCL: These devices required a manual configuration of both the "Autostart" function and the "Unused Apps" permission management to ensure the longevity of the sensing services.
- Samsung: While these devices generally required less manual intervention for autostart, specific audits of the "Unused Apps" permission settings were mandatory to avoid the automatic revocation of sensors during periods of low interaction.

If systematic diagnostic steps failed to restore data flow, the protocol dictated an immediate escalation to the technical team for deep-level debugging, ensuring that the period of data loss is kept to a minimum.

6 Supplementary Data – Restoring the monitoring of steps after the Google Fit API dismissal

In the following, we provide the description of the steps needed to address the issue raised from the Google Fit API dismissal while the BD4QoL study was underway. Custom software components were developed to address this difficulty, which led to a new release of the apps. Below, we describe the steps taken by the research team to restore the remote monitoring function of the BD4QoL apps.

- **System Cleanup:** Participants were first guided, during videoconference sessions, to uninstall the legacy version (v.2.14) of the Foreground App either through the long-press shortcut or via the smartphone's "Apps Management" settings to avoid software conflicts.
- **Guided Update:** The new version (v.2.15) was deployed through the BD4QoL Main App's notification bar or via a dedicated "Update Foreground App" option within the user profile section.
- **Managing Security Warnings:** During the APK installation, researchers assisted participants in navigating Android's security warnings regarding "harmful .apk" files, providing the necessary consent to proceed with the custom installation.
- **Full Reconfiguration:** The final step involved a comprehensive re-optimization of the app's environment, including:
    - Re-authenticating with participant credentials.
    - Granting persistent permissions for GPS location (Allow all the time) and Physical Activity tracking.
    - Manually disabling battery restrictions to ensure continuos background data collection.
    - Disabling the Android "Remove permissions and free up space" feature for unused apps.
    - Enabling the "Autostart" function where available.
    - Executing a "Force Upload" test to verify that the technical team could immediately confirm the upload of data to the study database.
